# Transcriptional effects of CRP* expression in *Escherichia coli*

**DOI:** 10.1186/1754-1611-3-13

**Published:** 2009-08-24

**Authors:** Reza Khankal, Jonathan W Chin, Debashis Ghosh, Patrick C Cirino

**Affiliations:** 1 Department of Chemical Engineering, The Pennsylvania State University, University Park, PA 16802, USA; 2 Department of Statistics, The Pennsylvania State University, University Park, PA 16802, USA

## Abstract

**Background:**

*Escherichia coli *exhibits diauxic growth in sugar mixtures due to CRP-mediated catabolite repression and inducer exclusion related to phosphotransferase system enzyme activity. Replacement of the native *crp *gene with a catabolite repression mutant (referred to as crp*) enables co-utilization of glucose and other sugars in *E. coli*. While previous studies have examined the effects of expressing CRP* mutants on the expression of specific catabolic genes, little is known about the global transcriptional effects of CRP* expression. In this study, we compare the transcriptome of *E. coli *W3110 (expressing wild-type CRP) to that of mutant strain PC05 (expressing CRP*) in the presence and absence of glucose.

**Results:**

The glucose effect is significantly suppressed in strain PC05 relative to strain W3110. The expression levels of glucose-sensitive genes are generally not altered by glucose to the same extent in strain PCO5 as compared to W3110. Only 23 of the 80 genes showing significant differential expression in the presence of glucose for strain PC05 are present among the 418 genes believed to be directly regulated by CRP. Genes involved in central carbon metabolism (including several TCA cycle genes) and amino acid biosynthesis, as well as genes encoding nutrient transport systems are among those whose transcript levels are most significantly affected by CRP* expression.

We present a detailed transcription analysis and relate these results to phenotypic differences between strains expressing wild-type CRP and CRP*. Notably, CRP* expression in the presence of glucose results in an elevated intracellular NADPH concentration and reduced NADH concentration relative to wild-type CRP. Meanwhile, a more drastic decrease in the NADPH/NADP^+ ^ratio is observed for the case of CRP* expression in strains engineered to reduce xylose to xylitol via a heterologously expressed, NADPH-dependent xylose reductase. Altered expression levels of transhydrogenase and TCA cycle genes, among others, are consistent with these observations.

**Conclusion:**

While the simplest model of CRP*-mediated gene expression assumes insensitivity to glucose (or cAMP), our results show that gene expression in the context of CRP* is very different from that of wild-type in the absence of glucose, and is influenced by the presence of glucose. Most of the transcription changes in response to CRP* expression are difficult to interpret in terms of possible systematic effects on metabolism. Elevated NADPH availability resulting from CRP* expression suggests potential biocatalytic applications of crp* strains that extend beyond relief of catabolite repression.

## Background

*E. coli *growing in a mixture of sugars exhibits diauxic growth characteristics, whereby glucose is preferentially assimilated before other sugars. This is due to CRP-mediated catabolite repression and inducer exclusion related to phosphotransferase system enzyme activity. It is well established that cyclic AMP (cAMP) and its receptor protein (CRP) are involved in transcriptional activation of catabolic genes [1,2], but the details of catabolite repression and inducer exclusion mechanisms and their relation to the levels of cAMP and CRP (also known as CAP) are not clear and have motivated many studies [3-7].

Inducer exclusion is a result of dephosphorylation of enzyme IIA^Glc ^of PTS [8,9] and catabolite repression is associated with altered levels of cAMP [10-12] and CRP [13]. Enzyme IIA^Glc^, when unphosphorylated, inhibits activity of other transport systems (non-PTS transporter) [14-16]. In its phosphorylated form, enzyme IIA^Glc ^stimulates adenylate cyclase activity, resulting in higher intracellular levels of cAMP [17,18] and the cAMP-CRP complex (global transcription activator).

Efforts to study or alleviate catabolite repression mediated by CRP have resulted in a series of CRP mutants isolated from strains lacking adenylate cyclase and having an apparent reduced dependence on cAMP for activating catabolic genes (called CRP*, CRP-in or CAP^c^) [1,19,20]. Genetically different crp* strains reported are also phenotypically different, showing different sensitivities to cyclic nucleotides and relieving catabolic repression of select genes examined to different extents [21]. For example, six different crp* mutants isolated after UV treatment and selection for a lactose^+ ^phenotype in an adenylate cyclase-deficient *E. coli *strain showed a variety of utilization patterns for different sugars (lactose, maltose, arabinose, xylose, ribose, mannose, mannitol) as well as different levels of activation of the *lac *operon by cAMP or cGMP [22]. Similar examples have been reported by others [3,21].

Ability to co-utilize sugars via relief of catabolite repression during microbial production of value-added chemicals has potential to improve bioproduction process economics [23]. We previously engineered *E. coli *to produce xylitol from xylose while metabolizing glucose as a source of carbon and energy (xylose metabolism is disabled) [24,25]. Expression of CRP* was an effective approach to promote expression of xylose transporters and enhance xylitol production in the presence of glucose. Although plasmid-based, CRP-independent expression of xylose transporters in wild-type *crp *strains also enhances xylose uptake and xylitol production in the presence of glucose [25], the favorable effects of CRP* expression were found to go beyond improving xylose transport and to include other beneficial phenotypes such as improved xylitol titer in controlled batch fermentation and reduced acetate production and higher yields on xylose reduced per mole of glucose consumed in resting cell transformations [26].

While CRP*s have been studied at the molecular level and the effects of expressing CRP* mutants on the expression of specific catabolic genes have been reported, the global transcriptional effects and regulatory consequences of CRP* expression is not known. Here, we report the results of comparisons between the transcriptome of *E. coli *W3110 (expressing wild-type CRP) and that of mutant strain PC05 (expressing CRP*) in the presence and absence of glucose through microarray analysis. Our results show that gene expression in PC05 is drastically different from that of W3110 in both the presence and absence of glucose, and that while expression of the CRP* allele used in this study has the general effect of suppressing transcriptional changes due to glucose, a significant response to glucose nonetheless remains. Results are analyzed in light of the observed differences between wild-type and CRP* strains during xylitol production. We identify many genes showing differential expression that are consistent with the observed elevated levels of glucose oxidation and NADPH-dependent xylose reduction for PC05 compared to W3110. A subsequent intracellular cofactor analysis reveals CRP*-correlated effects on cofactor levels that are consistent with the observed expression changes.

## Results

The *E. coli *W3110-derivative CRP* strains used in our studies are derived from *E. coli *donor strain ET25 [8], which expresses a CRP* mutant with three amino acid substitutions (I112L, T127I, and A144T) identical to those found in an earlier characterized CRP* strain CA8404 [1]. Amino acid position 127 lies in the cAMP binding pocket, and T127I or T127L mutations occur frequently in CRP* alleles [21,27], presumably serving to reduce the cAMP requirement to form an activating CRP complex [27]. Mutation A144T is also frequently found in different CRP* alleles [3,21,28] and can exhibit the CRP* phenotype to some extent even as the only mutation in the protein [29]. This position lies in the DNA binding domain of CRP and is suggested to improve affinity of the protein for CRP binding sites [29]. A fourth base substitution in the crp* sequence results in a T28K mutation, which is the result of native differences in the *crp *sequence between W3110 and the donor strain.

### Genome-wide transcriptional effects of glucose and CRP*

**Figure 1:**
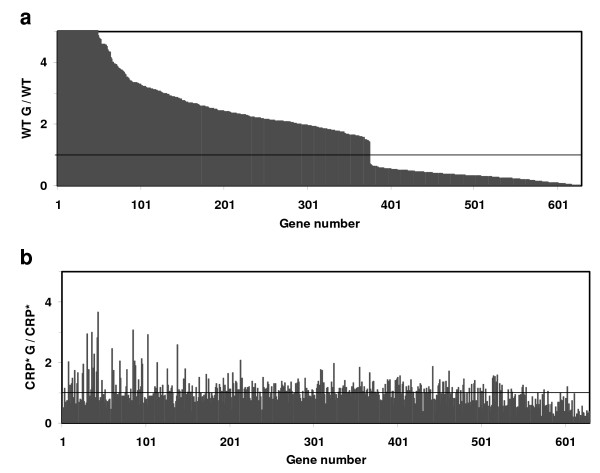
**Genome-wide transcriptional effects of glucose in strain W3110 expressing wild-type CRP, presented as expression ratios for individual genes showing significant differential expression in the presence and absence of glucose (WT G/WT)**. **a) **629 genes show significant changes in expression level in response to the presence of glucose in strain W3110. **b) **The changes in expression levels of the same genes shown in **(a) **in CRP* strain PC05, in response to glucose. Gene names and expression levels are given in supplementary Table S2 (see Additional file 2).

Table 1 summarizes the genome-wide effects of CRP* expression under the conditions tested, while Table 2 lists the average signal values and expression ratios for specific genes mentioned in this paper. Supplementary Table S1 (see Additional file 1) contains signal values for the complete probe set data for the *E. coli *K-12 genome. Transcriptome analysis of strain W3110 reveals that 629 genes show significant changes in expression level in response to the presence of glucose (comparison between WT G and WT in Figure 1a). 375 of these genes are upregulated by glucose, as depicted in Figure 1a. The complete list of expression levels of the genes that are differentially expressed between WT G and WT is provided in Supplementary Table S2 (see Additional file 2). Catabolic genes, membrane-related components, and sugar transporters (especially non-glucose PTS related enzymes) represent a large portion of genes repressed by glucose. In a study of CRP-dependent gene expression, Gosset and coworkers reported transcriptome analysis of CRP-dependent genes in another *E. coli *K-12 strain BW25113 [30]. In Table 3 we compare their result to results from our study for common conditions tested (i.e., WT G/WT). While their study did not examine CRP*, this comparison provides an indication of the consistency of glucose-responsive gene expression among different but similar strains. Our comparison focuses on genes involved in central metabolism and shows that the genes which are subject to glucose repression in BW25113 (such as *aceA *(isocitrate lyase monomer), *aldA *(aldehyde dehydrogenase A), *sdhA *(succinate dehydrogenase) and *sucA *(oxoglutarate dehydrogenase)) are also downregulated in the presence of glucose for W3110. However, not all the genes which are upregulated in the presence of glucose in BW25113 are upregulated in W3110 under the same conditions (examples are *aceE *(pyruvate dehydrogenase E1 component), *guaB *(IMP dehydrogenase), *rpsQ *(30S ribosomal subunit protein S17)). This is likely to be due to differences between these two strains [26,31-33] as well as the differences in experimental methods.

**Table 1:** Summary of genome-wide effects of CRP* expression.

Conditions compared	Number of genes showing significant differential expression	Up-regulated	Down-regulated	Genes in common with WT G/WT comparison
WT G/WT	629	375	254	629
CRP* G/CRP*	80	29	51	43
(CRP* G/CRP*)/(WT G/WT)	238	-----	-----	198
CRP* G/WT G	349	232	117	163
CRP*/WT	553	392	161	205
CRP* G/WT	481	330	151	218

Four different conditions were tested: W3110 in LB+glucose medium (WT G), W3110 in LB medium (WT), PC05 in LB+glucose medium (CRP* G), and PC05 in LB medium (CRP*). Differences in gene expression levels with a P-value of <0.05 were considered significant in each comparison.

**Table 2:** Expression levels for the genes discussed in this paper.

Gene	WT G	WT	CRP* G	CRP*	WT G/WT	CRP* G/CRP*	CRP* G/WT G	CRP* G/WT
*aceA*	1806	3171	2584	1980	0.6	1.3	1.4	0.8
*aceE*	7646	13291	10053	9167	0.6	1.1	1.3	0.8
*acs*	56	223	203	386	0.3	0.5	3.6	0.9
*aldA*	370	2471	937	1613	0.2	0.6	2.5	0.4
*argD*	44	38	73	158	1.1	0.5	1.7	1.9
*asnA*	594	1741	1409	1880	0.3	0.8	2.4	0.8
*cheR*	1588	2315	2311	1007	0.7	2.3	1.5	1.0
*cheY*	5365	6487	5893	2646	0.8	2.2	1.1	0.9
*citF*	50	25	306	269	2.0	1.1	6.1	12.2
*dhaM*	8790	1502	10556	3530	5.9	3.0	1.2	7.0
*edd*	4178	756	3582	980	5.5	3.7	0.9	4.7
*fruA*	3105	1289	5777	3659	2.4	1.6	1.9	4.5
*fruK*	3722	1934	6807	3866	1.9	1.8	1.8	3.5
*fumA*	956	3284	4013	3738	0.3	1.1	4.2	1.2
*gapC*	1316	581	2345	2383	2.3	1.0	1.8	4.0
*gatD*	1560	475	6080	7981	3.3	0.8	3.9	12.8
*gatR*	1776	786	6106	5039	2.3	1.2	3.4	7.8
*gcd*	491	467	171	67	1.1	2.5	0.4	0.4
*glnP*	2682	1037	1108	1374	2.6	0.8	0.4	1.1
*glpC*	564	8563	5595	13130	0.1	0.4	9.9	0.7
*glpF*	738	12693	2384	10944	0.1	0.2	3.2	0.2
*glpG*	1225	895	938	1426	1.4	0.7	0.8	1.1
*glpK*	360	8374	1579	8395	0.04	0.2	4.4	0.2
*glpQ*	159	4424	1747	9315	0.04	0.2	11.0	0.4
*glpT*	54	4285	893	9145	0.01	0.1	16.5	0.2
*gltB*	3219	2423	3388	1487	1.3	2.3	1.1	1.4
*gltD*	1881	1282	1829	595	1.5	3.1	1.0	1.4
*glyA*	1613	3148	3353	3448	0.5	1.0	2.1	1.1
*gntK*	631	217	670	259	2.9	2.6	1.1	3.1
*gntP*	87	354	321	799	0.2	0.4	3.7	0.9
*gntU*	334	98	260	126	3.4	2.1	0.8	2.7
*guaB*	4670	7698	2558	1733	0.6	1.5	0.6	0.3
*ldhA*	1823	1281	6014	3669	1.4	1.6	3.3	4.7
*maeB*	579	1420	1509	1839	0.4	0.8	2.6	1.1
*malF*	2381	2353	7365	9358	1.0	0.8	3.1	3.1
*manZ*	3993	2734	12806	9993	1.5	1.3	3.2	4.7
*mdh*	5878	9397	12603	15821	0.6	0.8	2.1	1.3
*mglA*	10	592	400	1205	0.02	0.3	40.0	0.7
*mglB*	38	1376	1529	4340	0.03	0.4	40.3	1.1
*nrfE*	783	21	531	754	36.5	0.7	0.7	24.7
*nrfF*	2460	41	1177	2267	59.7	0.5	0.5	28.6
*nrfG*	611	10	216	435	58.6	0.5	0.4	20.7
*pntA*	3946	6915	7400	7086	0.6	1.0	1.9	1.1
*pntB*	1736	2546	3057	3083	0.7	1.0	1.8	1.2
*ppsA*	247	6026	1325	3494	0.04	0.4	5.4	0.2
*proA*	2536	2397	13	12	1.1	1.1	0.01	0.01
*proB*	3589	3092	7	4	1.2	1.9	0.00	0.00
*ptsG*	13731	3926	16556	5403	3.5	3.1	1.2	4.2
*rpsQ*	6405	12505	9398	7652	0.5	1.2	1.5	0.8
*sdhA*	1033	7257	3292	3154	0.1	1.0	3.2	0.5
*sdhB*	895	5161	2407	2521	0.2	1.0	2.7	0.5
*sdhD*	556	5466	2568	1980	0.1	1.3	4.6	0.5
*serA*	2256	956	1638	793	2.4	2.1	0.7	1.7
*serC*	3904	2365	3230	1738	1.7	1.9	0.8	1.4
*srlA*	174	4601	313	3037	0.04	0.1	1.8	0.1
*sthA*	3236	3764	1936	2741	0.9	0.7	0.6	0.5
*sucA*	3988	8390	4822	4421	0.5	1.1	1.2	0.6
*tdcA*	74	4064	3412	8483	0.02	0.4	45.9	0.8
*tdcB*	147	4614	7708	13552	0.03	0.6	52.4	1.7
*tdcE*	95	397	3932	11414	0.2	0.3	41.4	9.9
*tdcG*	126	182	3018	14708	0.7	0.2	23.9	16.6
*thiC*	163	179	212	455	0.9	0.5	1.3	1.2
*thiE*	136	140	183	412	1.0	0.4	1.4	1.3
*thrA*	812	2686	1997	1279	0.3	1.6	2.5	0.7
*thrB*	849	2857	2165	1353	0.3	1.6	2.6	0.8
*thrC*	783	1722	1594	1378	0.5	1.2	2.0	0.9
*tnaA*	688	16366	10805	23343	0.04	0.5	15.7	0.7
*tnaB*	74	3825	1957	9810	0.02	0.2	26.6	0.5
*treB*	4465	17280	4711	19044	0.3	0.3	1.1	0.3
*treC*	3007	12164	2606	15416	0.3	0.2	0.9	0.2
*Upd*	11584	3965	4736	6483	2.9	0.7	0.4	1.2
*ymfL*	29	56	364	71	0.5	5.1	12.5	6.6
*ymfO*	114	177	961	137	0.7	7.0	8.4	5.4

Four different conditions were tested: W3110 in LB+glucose medium (WT G), W3110 in LB medium (WT), PC05 in LB+glucose medium (CRP* G), and PC05 in LB medium (CRP*)

**Table 3:** Comparison between expression levels (signal values) of the wild-type strain genes in response to the presence of glucose in two different studies.

Gene symbol	This study		Study by Gosset et al. [30]	
		
	WT G	WT G/WT	WT G	WT G/WT
Upregulated genes in the study by Gosset and coworkers				
*aceE*	7646	0.58	8177	4.4
*fis*	3058	0.99	5726	6.9
*guaB*	4670	0.61	2749	4.2
*ptsG*	13731	3.50	2387	3.2
*rplS*	8353	0.84	5704	2.7
*rpmE*	13204	1.18	8773	3.9
*rpsQ*	6405	0.51	3311	2.7
*rpsT*	10344	1.8	24476	2.6
*spf*	11308	3.40	26801	11.2
				
Downregulated genes in the study by Gosset and coworkers				
*aceA*	1806	0.57	498	0.2
*aceB*	1466	0.65	296	0.2
*aldA*	370	0.15	305	0.1
*fumA*	956	0.29	819	0.3
*gltA*	126	0.27	452	0.1
*mdh*	5878	0.62	996	0.2
*pckA*	6733	0.56	765	0.3
*sdhA*	1033	0.14	614	0.2
*sdhB*	556	0.10	401	0.2
*sucA*	3988	0.47	822	0.2
*sucB*	6087	0.60	802	0.1
*sucC*	5447	0.59	1250	0.2
*sucD*	3439	0.58	526	0.1

This comparison focuses on genes involved in central metabolism.

Figure 1b depicts the changes in expression levels of the same genes shown in Figure 1a for CRP mutant strain PC05 in response to glucose. The average WT G/WT ratio for the genes which are upregulated in W3110 in the presence of glucose is 4.06 while the average CRP* G/CRP* ratio for the same genes is 1.07. For downregulated genes in W3110 by glucose, WT G/WT and CRP* G/CRP* ratios are 0.32 and 0.81 respectively. These results show that genes whose expression is significantly altered by glucose in strain W3110 are generally not altered to the same extent in strain PCO5 and that CRP* suppresses this effect of glucose.

**Figure 2:**
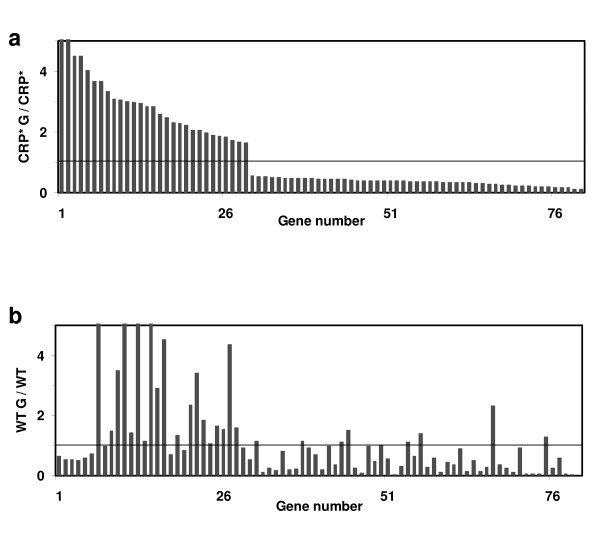
Genome-wide transcriptional effects of glucose in CRP* strain PC05, presented as expression ratios for individual genes showing significant differential expression in the presence and absence of glucose (CRP* G/CRP*). **a) **80 genes show significant changes in expression level for strain PC05. Only 29 are upregulated. **b) **The changes in expression levels of the same genes shown in **(a) **for strain W3110, in response to glucose. Gene names and expression levels are given in supplementary Table S3 (see Additional file 3).

Figure 2 depicts that fewer genes show significant changes in expression level for strain PC05 (80 genes) compared to W3110 (629 genes) when grown in the presence versus absence of glucose. 29 of these genes are upregulated in the presence of glucose. This confirms the expected role of CRP* in the alleviation of glucose repression. Only 43 genes are common between those of Figure 1 and Figure 2. In contrast to W3110, the number of genes that are repressed in the presence of glucose in PC05 is greater than the number of genes that are upregulated. Only 3% of genes that are upregulated in W3110 in the presence of glucose are also upregulated in PC05 in the same condition, while 12% of glucose-repressed genes in W3110 are also repressed by glucose in PC05.

The complete list of expression levels of the genes that are differentially expressed between in PC05 with versus without glucose is provided in Supplementary Table S3 (see Additional file 3). Specific examples of genes that are upregulated in PC05 in response to glucose include: the PTS gene *ptsG*, *che *genes (involved in regulation of chemotaxis), *dhaM *(associated with dihydroxyacetone kinase), *edd *(encoding phosphogluconate dehydratase of the Entner-Doudoroff pathway), *gnt *genes (gluconate transport and metabolism), genes involved in amino acid metabolism such as *glt (*glutamate synthase), and *ser *(serine biosynthesis) genes, and *ymf *genes of the lambdoid prophage element e14. Genes that are downregulated in PC05 in response to glucose include: *argD *(involved in lysine and arginine biosynthesis), *glp *genes (glycerol transport and metabolism), *gntP *(encoding a gluconate transporter), *srlA *(glucitol/sorbitol PTS system), *thiCE *genes (involved in thiamine biosynthesis), and *treBC *genes (trehalose transport and metabolism). Refer to Table 2 for expression levels and ratios.

418 genes in the *E. coli *genome are suggested to be regulated in part by CRP, as reported by the most current EcoCyc database [34]. While the modes of regulation of many of these genes are complicated and not well understood (often involving multiple transcription factor binding sites), the CRP-cAMP complex is assigned to be a transcriptional activator for approximately 321 genes (implying upregulation in the absence of glucose) and a repressor for approximately 46 genes (implying downregulation in the absence of glucose) (in some cases the role of CRP is dual or unclear). While 629 genes show significant changes in their expression levels in response to the presence of glucose in W3110 (Figure 1), only 19% of them (118 out of 629) have a CRP binding site close to their start codon (these genes are highlighted in green in Additional file 2 (Table S2)). This result is perhaps not unexpected when considering that most genes under CRP control are also regulated by other transcription factors [34]. Of these 118 genes, 25 are upregulated while 93 show reduced expression in the presence of glucose. 77 out of the 93 genes downregulated in glucose are described in Ecocyc as being activated by CRP-cAMP, showing good agreement with the expected inverse relationship between glucose presence and CRP-cAMP activity. Meanwhile only 23 of the 80 genes showing significant differential expression in the presence of glucose for strain PC05 are present among the list of 418 genes believed to be directly regulated by CRP (highlighted in green in Additional file 3 (Table S3); 7 are upregulated in glucose, and 13 out of the remaining 16 downregulated genes are reported to be activated by CRP-cAMP and therefore expected to show lower expression in the presence of glucose), again demonstrating significant alleviation of catabolite repression. Thus, the majority of expression changes resulting from the presence of glucose or the CRP* mutations are not directly related to altered regulation by CRP at CRP binding sites, but rather due to secondary effects resulting from a smaller number of direct, CRP-mediated expression differences.

**Figure 3:**
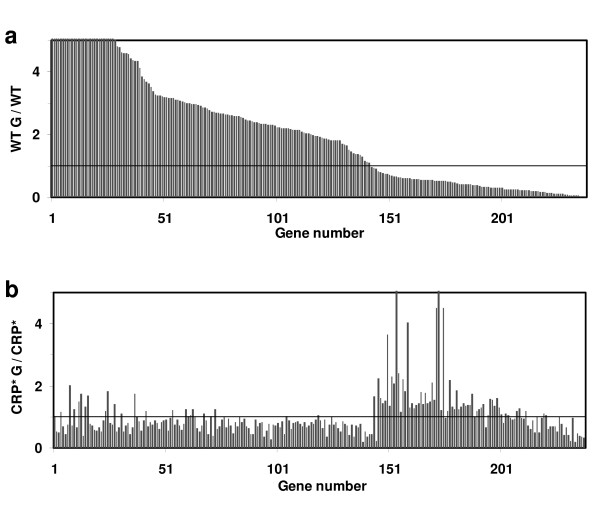
Genes that respond differently to the presence of glucose in W3110 compared to PC05, (CRP* G/CRP*)/(WT G/WT). **a) **Ratio of expression levels of these 238 genes in strain W3110 in the presence and absence of glucose (WT G/WT). **b) **Ratio of expression levels of the same genes shown in **(a) **in strain PC05, in the presence and absence of glucose (CRP* G/CRP*). Gene names and expression levels are given in supplementary Table S4 (see Additional file 4).

To investigate which genes respond differently to glucose in PC05 compared to W3110, an interaction term ((CRP* G/CRP*)/(WT G/WT)) was examined with the same criteria as pair-wise comparisons. This comparison reports the difference of differences and reveals that 238 genes respond differently to the presence of glucose in W3110 compared to PC05, as illustrated in Figures 3a and 3b (listed in supplementary Table S4 (see Additional file 4)). As shown in Table 2, *ppsA *(encoding phosphoenolpyruvate synthase), *glp *genes (involved in glycerol transport and metabolism), *mgl *genes of the galactose ABC transporter, and TCA cycle genes *fumA *(fumarate hydratase class I) and *sdhA, sdhB, sdhD *(succinate dehydrogenase) all show significantly different responses to glucose in W3110 compared to PC05.

**Figure 4:**
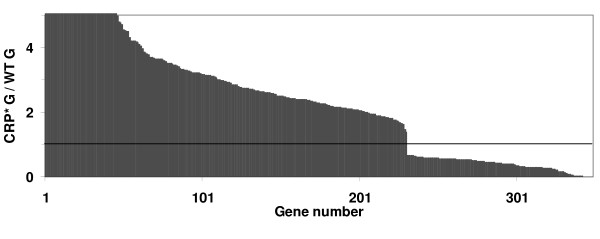
Significant genome-wide transcription effects of expressing CRP* (strain PC05) instead of wild-type CRP (W3110) in the presence of glucose, presented as individual gene expression ratios (CRP* G/WT G). 349 genes show significant changes in their expression levels between CRP* G and WT G conditions. Gene names and expression levels are given in supplementary Table S5 (see Additional file 5).

Pair-wise comparison between the CRP* G and WT G conditions identifies the changes in transcriptional levels of genes affected by CRP* in the presence of glucose. In this category, 349 genes show significant changes in their expression levels (Table 1), as depicted in Figure 4 and as listed in supplementary Table S5 (see Additional file 5). Many of the genes upregulated by CRP* (strain PC05) are involved in transport, catabolism, and amino acid metabolism (Table 2), including *glp *genes (involved in glycerol transport and metabolism), *mgl *genes (galactose ABC transporter), *tdc *genes (involved in serine and threonine metabolism), and *tnaB *(encoding a tryptophan transporter). Genes downregulated by CRP* in this comparison include *gcd *(encoding glucose dehydrogenase), *glnP *(glutamine ABC transporter), *pro *genes (involved in proline biosynthesis), and *udp *(uridine phosphorylase, involved in pyrimidine ribonucleoside metabolism). Additional differentially expressed genes of relevance to this study are described in the Discussion.

Pair-wise comparison of gene expression in PC05 and W3110 grown on LB (without glucose) reveals changes in gene transcription levels as a result of the CRP* mutation. This comparison shows that 553 genes are expressed differently (392 of them are upregulated) between these two strains in the absence of glucose. These results are summarized in Table 1, and the specific genes are listed in supplementary Table S6 (see Additional file 6). *gapC *(encoding glyceraldehyde 3-phosphate dehydrogenase), *nrf *genes (involved in anaerobic respiration), and *tdc *genes (involved in serine and threonine metabolism) are examples of genes upregulated by CRP*.

Finally, to examine the extent to which CRP* reduces the glucose effect, we performed a pair-wise test between two conditions: PC05 in the presence of glucose (CRP* G) and W3110 in the absence of glucose (WT). Our results show that the transcriptional levels of 481 genes are significantly different between these two conditions. These results are summarized in Table 1 and the specific genes and expression values are listed in supplementary Table S7 (see Additional file 7). Examples of genes most significantly upregulated by CRP* in glucose include the *nrf *genes, *citF *(encoding citrate lyase), *gat *genes (involved in hexitol transport and metabolism), *edd *(encoding phosphogluconate dehydratase), *ldhA *(lactate dehydrogenase), *fru *genes (fructose transport and metabolism), *manZ *(mannose PTS permease), *ptsG*, and *malF *(maltose ABC transporter).

### Real-Time Reverse Transcription PCR

To confirm the microarray results, the transcript levels of *ppsA *and *pntA*, both of which showed significant changes in their transcriptional levels under the various conditions tested, were compared by real-time reverse transcription PCR. The transcript of *rpsQ *was also analyzed, as this gene was noted to respond quite differently to the presence of glucose with wild-type CRP in our study as compared to the study by Gosset (WT G/WT value of 0.51 compared to 2.7) [30]. Data are presented in supplementary Table S8b as fold-changes (signal ratios) for all conditions tested, and show a good agreement between microarray and real-time RT-PCR results (see Additional file 8).

### Cofactor analysis

In order to better understand how CRP* expression may influence NADPH availability for xylose reduction, intracellular cofactor concentrations were quantified for wild-type and CRP* strains engineered to produce xylitol. To prevent xylose metabolism in these strains, *xylB *encoding xylulokinase was deleted [24]. The wild-type CRP strain (W3110*ΔxylB*) was transformed with plasmid pPCC207 for inducible co-expression of an NADPH-dependent xylose reductase (CbXR) and the *E. coli *ATP-dependent xylose transporter system (XylFGH) [25], while the CRP* strain (PC05*ΔxylB*) was transformed with plasmid pLOI3815 for CbXR expression [24]. The strains were then compared after growth on glucose (no xylitol production) versus growth on glucose plus xylose (resulting in xylose reduction).

Results from the intracellular cofactor concentration measurements are summarized in Table 4. Also listed are the xylitol production results for these strains in batch fermentations and resting cell cultures. Note that the CRP* strain produced considerably more xylitol and with a higher yield. In the absence of xylose, the NADPH concentration is significantly higher in the CRP* strain (0.8 versus 0.5 μmol (g cdw)^-1^). Meanwhile, given the ability to reduce xylose to xylitol, the NADPH concentration falls to a much lower level in the CRP* strain (from 0.8 to 0.1 μmol (g cdw)^-1^) compared to wild-type CRP (from 0.5 to 0.3 μmol (g cdw)^-1^). While the NADPH/NADP^+ ^ratios are nearly identical for both strains in the absence of glucose, the significant consumption of NADPH during xylose reduction coincides with a significantly larger drop in the NADPH/NADP^+ ^ratio for CRP* (from 0.09 to 0.01) compared to wild-type CRP (from 0.10 to 0.05). Also note that the oxidized NADP^+ ^concentrations are significantly elevated in the CRP* strain, while both NADH and NAD^+ ^concentrations are much lower. The net effects are higher total NADP(H) (i.e. NADPH plus NADP^+^) concentrations and lower NAD(H) concentrations in the CRP* strains compared to wild-type. It is also noteworthy that the NADH/NAD^+ ^ratio is significantly lower in the CRP* strain under both conditions tested.

**Table 4:** Culture performance and intracellular cofactor levels and ratios for strains engineered to produce xylitol.

	Batch fermentation^a^		Resting cells^b^		Cofactor concentrations μmol (g cdw)^-1^				Cofactor ratios	
	Glucose consumed (mM)	Xylitol produced (mM)	Acetate produced (mM)	Xylitol yield^c^	NADH	NAD^+^	NADPH	NADP^+^	NADH/NAD^+^	NADPH/NADP^+^
W3110Δ*xylB *+ pPCC207, **GX**	248	223	19	2.5	10.3	47.9	0.3 ± 0.05	5.8	0.22	0.05
W3110Δ*xylB *+ pPCC207, **G**	-	-	-	-	10 ± 1.8	47.9	0.5	5.1 ± 1.0	0.21	0.10
PC05Δ*xylB *+ pLOI3815, **GX**	301	680	1.3 ± 1.4	3.9	1.8 ± 0.7	33.1	0.1 ± 0.04	9.9 ± 2.3	0.05	0.01
PC05Δ*xylB *+ pLOI3815, **G**	-	-	-	-	3.8 ± 0.7	39.2	0.8	8.7 ± 2.6	0.10	0.09

W3110*ΔxylB *harboring plasmid pPCC207 expresses CbXR and XylFGH, while PC05*ΔxylB *harboring plasmid pPLOI3815 expresses CbXR. Strains were grown in the presence of glucose only (G) or in glucose plus xylose (GX), enabling xylose reduction during glucose metabolism. Standard deviations were less than 15% unless noted.

^a ^Reported in Table 4 of reference [26].

^b ^Resting cells were prepared as described [61]. Values reported were measured after 24 hours of biotransformation with cells suspended to OD_600 _= 2.0.

^c ^Reported as moles xylitol produced per mole glucose consumed.

## Discussion

We previously used a CRP* strain to promote expression of xylose transporters in the presence of glucose to produce xylitol from a glucose+xylose mixture, with xylose metabolism disabled [24]. Plasmid-based, CRP-independent expression of xylose transporters in wild-type *crp *strains was an alternative strategy we explored to enhance xylose uptake and xylitol production in the presence of glucose [25]. However the favorable effects of CRP* expression were found to go beyond improving xylose transport and to include other beneficial phenotypes such as reduced acetate production and higher yields on xylose reduced per mole of glucose consumed (as shown in Table 4) [26].

Transcription changes associated with CRP* expression are extensive. While many of the genes known to be regulated by CRP show altered expression in the context of CRP*, the majority of differentially expressed genes are not known to be directly under CRP control. The complex network of genes showing altered regulation due to secondary effects of CRP* is not likely to be metabolically systematic, since the mutant CRP used in this study (i.e. CRP*) does not have an evolved physiological role, was isolated under very particular growth conditions, and does not simply serve as a constitutive, cAMP-independent regulator. Therefore the difficulty in identifying clear patterns of differential expression of metabolically related genes is perhaps not surprising (the mutations in CRP* may not uniformly alter the regulator's natural physiological role at different control sites). Rather than attempting to assign physiological meaning to the altered transcriptome, we instead identify gene expression changes that help to explain the beneficial effects of CRP* expression as they relate to xylitol production. Specifically, we focus on genes that may affect NADPH availability.

### Improved cofactor availability for xylitol production

In *E. coli*, complete oxidation of glucose during aerobic growth requires that respiration and anabolic metabolism consume reducing equivalents as they are generated [35]. Elevated glucose flux beyond the capacity of respiration and growth results in incomplete oxidation and acid secretion. Heterologous, NADPH-dependent xylitol production can act as an added electron sink in strains producing xylitol from a mixture of glucose and xylose, and an increased ability to produce xylitol during aerobic growth is expected to increase glucose oxidation and tricarboxylic acid (TCA) cycle flux, provided reducing equivalents from NADH can be converted to NADPH. Alternately, increased expression of genes involved in glucose oxidation would allow for increased xylitol production, provided reducing equivalents can be delivered as NADPH. Our transcription analysis sheds light on the observed ability of CRP* strains expressing xylose reductase to produce more xylitol and secrete less acetate than similar wild-type CRP strains constitutively expressing a xylose transporter.

TCA cycle genes involved in reactions between succinate and oxaloacetate are upregulated in PC05 compared to W3110 in the presence of glucose, as listed in Table 2. These include *sdhA*, *sdhB*, *sdhD *(encoding succinate dehydrogenase), *fumA *(encoding fumarate hydratase class I), and *mdh *(encoding malate dehydrogenase). All of these genes are known to have CRP binding sites in their promoter regions, so increased expression in PC05 is likely due to direct regulatory effects of CRP*. A strain with a more active TCA cycle potentially increases glucose oxidation, produces more NADPH and produces less acetate [36,37]. Upregulation of *acs *(acetyl-CoA synthetase) in CRP* G compared to WT G (3.63-fold) may promote acetate assimilation instead of accumulation. Also shown in Table 2, *sthA *encoding soluble pyridine nucleotide transhydrogenase is differentially expressed in CRP* G compared to WT G (CRP* G/WT G ratio of 0.6). SthA is believed to primarily oxidize NADPH to regenerate NADH and an increase in NADPH demand corresponds to reduced *sthA *expression [38,39]. As shown in Table 2, *sthA *expression is lower with CRP* G compared to WT G. Transcriptional control of *sthA *is not well understood, but the lack of an apparent CRP binding site suggests that reduced *sthA *expression may be a result of increased NADPH demand rather than a direct result of CRP*-mediated control. Consistent with this apparent elevated demand for NADPH is the fact that the genes encoding both subunits of the membrane-bound, proton-translocating pyridine nucleotide transhydrogenase (*pntA *and *pntB*) are upregulated in CRP* G compared to WT G (1.88 and 1.76-fold respectively). PntAB has been reported to produce 35–45% of the NADPH required for *E. coli *biosynthesis during aerobic growth [38]. Interestingly, the *maeB *gene encoding NADP-linked malic enzyme (decarboxylating malate to pyruvate) can serve as another source of NADPH regeneration in *E. coli *and also shows a higher level of transcription in PC05 compared to W3110 in the presence of glucose (CRP* G/WT G ratio of 2.6). Changes in transcriptional patterns of the above mentioned genes can be in response to increased demands for NADPH in CRP* strains, perhaps as this relates to apparently increased anabolic demands (described below).

To ensure continued growth, *E. coli *balances intracellular concentrations and ratios of the reduced and oxidized cofactors through a complex interplay between catabolic metabolism, anabolic metabolism, redox-sensitive regulation (both genetic and allosteric) and transhydrogenase activities [35,40-43]. While the mechanisms of maintaining redox balance are not well understood, the expression levels of enzymes and regulators involved in redox metabolism play a critical role. It is thus perhaps not surprising that altering the activity of a global regulator has a significant impact on cofactor concentrations and the range of attainable redox states (as demonstrated in Table 4). NADH regulates the activity of a number of enzymes involved in central metabolism and glucose oxidation (e.g. pyruvate dehydrogenase [44,45], citrate synthase [46,47] and α-ketoglutarate dehydrogenase [48]). CRP* expression results in reduced NADH levels, increased production of NADPH relative to NADH, and increased tolerance to a range of NADPH levels and NADPH/NADP^+ ^ratios, all of which are likely to improve NADPH-dependent xylitol production during glucose metabolism.

### Expression of "unnecessary" genes

Most of the genes that are differentially expressed between PC05 and W3110 in the presence of glucose (231 out of 349) are upregulated with CRP* (Figure 4), supporting the generally assumed behavior of CRP* in alleviating glucose-dependent catabolite repression. The otherwise unnecessary upregulation of these genes likely causes a significant increase in demand for carbon and energy, helping to explain the slower growth rate observed for PC05 compared to W3110 [24]. Notable genes that fall into this upregulated category include many involved in amino acids metabolism, such as *tnaA *(encoding tryptophanase), *thrA *(aspartokinase I and homoserine dehydrogenase I), *glyA *(serine hydroxymethyl transferase), *tdcB *(threonine dehydratase), *thrC *(threonine synthase), *thrB *(homoserine kinase), and *asnA *(asparagine synthetase A) (refer to Table 2). Upregulation of amino acid metabolism pathways may be in response to increased protein synthesis demands caused by upregulation of other genes.

### Catabolite repression and inducer exclusion

A relationship between the phosphorylation state of enzyme IIA^Glc ^and the intracellular "phosphoenolpyruvate (PEP)/pyruvate" ratio has been suggested [9]. Decreased levels of phosphorylated enzyme IIA^Glc ^is usually accompanied by decreased PEP/pyruvate ratios. The crucial role of the unphosphorylated form of enzyme IIA^Glc ^in catabolite repression and inducer exclusion is well documented [8,9,14-18]. As shown in Table 2, phosphoenolpyruvate synthase (encoded by *ppsA*) is expressed to a higher level in PC05 compared to W3110 in the presence of glucose (CRP* G/WT G ratio of 5.35). Upregulation of this enzyme which mediates conversion of pyruvate to PEP may increase the intracellular PEP/pyruvate ratio, resulting in an increase in the phosphorylated form of enzyme IIA^Glc^. This in turn may increase adenylate cyclase activity [17,18] and further help to alleviate catabolite repression and inducer exclusion in PC05.

## Conclusion

We have used microarray analysis to compare the transcriptomes of *E. coli *W3110 expressing wild-type CRP and mutant strain PC05 expressing CRP* in the presence and absence of glucose. Table 1 summarizes the genome-wide effects of CRP* expression under the conditions tested. Gene expression in the context of CRP* in the presence of glucose is very different from that of wild-type in the absence of glucose. Although fewer genes show expression sensitivity to glucose in PC05 compared to W3110, CRP* does not completely eliminate glucose effects. As expected, CRP* expression causes increased expression of genes involved in nutrient transport and catabolism (among many others). In addition, several genes showing significant differential expression in CRP* versus wild-type CRP help to explain the observed differences in cofactor levels and metabolic behavior of CRP* strains used in xylitol production.

## Materials and methods

### General

*E. coli *K-12 strain W3110 (ATCC 27325) and its derivatives were maintained on plates containing Luria-Bertani (LB) medium (10 g tryptone, 5 g yeast extract, 5 g NaCl, and 15 g agar per liter). Methods for construction of strains PC05 (W3110 and crp*), PC07 (W3110Δ*xylB*), and PC09 (PC05Δ*xylB*) were described previously [24]. Briefly, the crp* gene and *xylB *deletion were introduced into W3110 via P1 phage transduction using a lysate from strain ET25 (crp*::Tn10) [8] and PC06 (W3110, Δ*xylB*::FRT-*aac*-FRT) [24] followed by selections on tetracycline (for crp*) or apramycin (for Δ*xylB*) plates. Plasmid pLOI3815 is a medium copy, pBR322-origin vector carrying a kanamycin resistance marker and the xylose reductase gene from *Candida boidinii*, which is located downstream of *tac *promoter and upstream of a transcription termination sequence [24]. Xylose transporter genes *xylFGH *(ATP-dependent xylose transporter system) were cloned downstream of CbXR in pLOI3815 to make plasmid pPCC207.

Amino acid substitutions in the CRP* were confirmed by sequencing. The crp* phenotype was verified in two ways. First, several Tet^R ^transductants were grown in LB medium containing glucose (1%) and xylose (1%). Cells were harvested at mid logarithmic growth phase and washed twice in phosphate buffer containing kanamycin (50 μg/mL). After allowing time for residual sugars to be cleared, the cells were resuspended a final time in buffer containing xylose (1%), kanamycin, and 1% triphenyltetrazolium chloride (TTC). Reduction of TTC results in red color formation and indicates constitutive xylose utilization. The crp* phenotype was additionally confirmed using HPLC to verify simultaneous glucose and xylose consumption in batch cultures [24].

### Growth conditions

Four different conditions were tested in this study: W3110 in LB medium (WT), W3110 in LB+glucose medium (WT G), PC05 in LB medium (CRP*), and PC05 in LB+glucose medium (CRP* G). All experiments were performed at least in triplicate and all data reported are the average of at least three experiments. Cell culture optical density was measured at 600 nm (OD_600_) using a SPECTRAMax PLUS^384 ^spectrophotometer (Molecular Devices). Cells grown for harvesting were prepared briefly as follows. Overnight pre-seed cultures were prepared by inoculating 3 ml of LB medium (in 13 × 100 mm tube) with a few colonies from a fresh LB plate. The overnight cultures were used to inoculate, to an OD_600 _of 0.1, 50 ml LB media (with or without 0.4% glucose supplementation) seed cultures in a 250 ml shake-flask. The seed culture were grown at 37°C to an OD_600 _of ~2 and then were used to directly inoculate, to an OD_600 _of 0.02, 100 ml LB media (with or without 0.4% glucose supplementation) cultures in a 500 ml flask. These cultures were grown at 37°C and 250 rpm to an OD_600 _of 0.5.

### Cell harvesting and preparation of RNA

Cells from the 100 ml culture were harvested at an OD_600 _of 0.5 (early logarithmic growth phase) by immediately placing on ice, transferring to 50 ml falcon tubes and centrifuging at 4°C for 5 minutes before treating with lysozyme. Promega PureYield™RNA Midiprep System kit was used for RNA extraction. As a preliminary check, RNA yield and quality were determined by spectrophotometry according to the manufacturer's protocol and the integrity of the purified RNA was determined by formaldehyde agarose gel electrophoresis.

### Labeling, hybridization and scanning

Total RNA concentration and purity were determined using a NanoDrop spectrophotometer and total RNA integrity was examined using an Agilent Bioanalyzer. Total RNA of sufficient concentration, purity, and integrity was labeled and subsequently hybridized to Affymetrix GeneChip microarrays by the Penn State DNA Microarray Facility according to the manufacturer's instructions (Affymetrix Inc, Santa Clara, CA). Briefly, 10 μg of total RNA was converted to cDNA using random primed reverse transcription. cDNA was purified by removing the RNA via hydrolysis with NaOH and then neutralizing the solution. Purified cDNA was fragmented and subsequently end-labeled with biotin. Fragmented, end-labeled cDNA was dissolved in hybridization cocktail and hybridized to Affymetrix GeneChip *E. coli *Genome 2.0 Arrays (approximately 10000 probe set) for 16 hours at 45°C. The details of GeneChip *E. coli *Genome 2.0 Arrays are described by Affymetrix [49].

After hybridization, the hybridization cocktail was removed and the arrays were washed to remove unbound and non-specifically bound cDNA. Hybridization was detected by staining the arrays with streptavidin phycoerythrin. All washing and staining was performed using the Affymetrix GeneChip Fluidics Station 450 according to the manufacturer's instructions (Affymetrix Inc, Santa Clara, CA). Stained arrays were scanned using the Affymetrix GCS3000 7G scanner.

### Microarray data analysis

A minimum of three data sets was generated for each of the four different conditions tested (based on the combination of the strains W3110 and PC05 in LB and LB+glucose media). Affymetrix Expression Console™software (Version 1.1) was used for background adjustment, normalization and summarization of chip level data in the form of feature intensity (CEL) files in order to generate probe set summarization (CHP) files, using the probe logarithmic intensity error (PLIER) method. Data from CHP files were then exported to a Microsoft Excel spreadsheet for further analysis. Signal values for 10208 probsets from GeneChip *E. coli *Genome 2.0 Arrays were filtered to extract probe set data for only the *E. coli *K-12 strain. All calculations and analyses were performed on the 4070 genes remaining after filtration.

Signal values were transformed to the log base for the pair-wise comparisons. A linear model was fitted to each gene using the Bioconductor software package LIMMA [50,51] in the R environment [52]. The linear model coefficients were used to calculate significant differences in expression levels for all pair-wise comparisons. The P-values were adjusted by the Benjamini-Hochberg method [53] and genes with a P-value of <0.05 were considered as those with significantly different expression levels under different conditions tested. Data are reported as expression levels (signal values) or ratios of expression levels. Supplementary Table S1 (Additional file 1) contains signal values for the complete probe set data for the *E. coli *K-12 genome. Written code in R [52] (file name: sup_code.doc) can be found in supplementary material (see Additional file 9). Gene Annotations were transformed from AFFY probe set ID's to Entrez gene IDs using NETAFFX on the Affymetrix website [49]. The online database for annotation, visualization and integrated discovery (DAVID) [54,55] and Kyoto Encyclopedia of Genes and Genome (KEGG) [56] were used for pathway visualization and gene ontology (GO) classification.

### Real-time, Reverse Transcription PCR

Total RNA samples were isolated the same way as for microarray studies. *ppsA *(phosphoenolpyruvate synthase), *pntA *(membrane-bound proton-translocating pyridine nucleotide transhydrogenase), and *rpsQ *(30S ribosomal subunit protein S17) were selected for confirmation by real-time reverse transcription PCR with *rrsH *(encoding 16S ribosomal RNA) as a control. Primer and probe sequences used for RT-PCR are listed in supplementary Table S8a (Additional file 8) and were designed by Deborah S. Grove of the Penn State Nucleic Acid Facility using Primer Express v2.0 (Applied Biosystems, Foster City, CA). Probes were synthesized by Biosearch (Novato, CA). The Applied Biosystems High Capacity cDNA Reverse Transcription Kit (part number 4368813) was used for reverse transcription according to the manufacturer's instructions for cDNA production. cDNA was amplified in an ABI 7300 real-time machine using TaqMan^® ^Universal PCR Master Mix, No AmpErase^® ^UNG (part number 4324018). Output was analyzed using the  method [57].

### Cofactor measurements

The cofactor analysis used in this study is based on the methods developed by Bernofsky and Swan [58], and modified by Gibon and Larher [59], and Walton and Stewart [60]. To investigate the effect of xylitol production on intracellular NADP(H) and NAD(H) levels, cofactor concentrations and ratios were measured and compared in PC05Δ*xylB *strain harboring pLOI3815 and W3110Δ*xylB *harboring pPCC207. Seed cultures were grown at 37°C to an OD_600 _of ~2 and then were used to directly inoculate, to an OD_600 _of 0.02, 100 ml LB medium supplemented with 100 mM glucose, 100 mM xylose (or 200 mM glucose for non-xylitol producing conditions), 50 mM MOPS, kanamycin monosulfate (50 μg/ml) and isopropyl-B-D-thiogalactopyranoside (IPTG, 100 μM) in a 500 ml flask. These cultures were grown at 30°C and 250 rpm to an OD_600 _of 0.5. Cells were immediately chilled on ice and harvested by pelleting (4°C, 15 min, 3750 rpm) to achieve a final OD_600 _of 30 in 1 ml. To isolate the oxidized forms, the pellet was resuspended in 0.5 ml of 0.3 M HCl, 50 mM Tricine-NaOH (pH 8.0). To isolate the reduced forms, the pellet was resuspended in 0.5 ml of 0.3 M NaOH. All samples were then heated to 60°C for 7 minutes followed by a neutralization step (0.5 ml 0.3 M NaOH for oxidized forms, 0.3 ml 0.3 M HCl, 0.2 ml 1.0 M Tricine-NaOH (pH 8.0) for reduced forms). The neutralized solutions were then centrifuged (4°C, 60 min, 13000 rpm) and the supernatants were transferred to a new microcentrifuge tube.

Cofactor levels were measured in a 96-well microtiter plate. Either 40 μl of oxidized sample and 40 μl 0.1 M NaCl, or 80 μl of reduced sample was aliquoted to a single well. The 2X stock solution of the reaction mixture consisted of equal volumes of 1.0 M Tricine-NaOH (pH 8.0), 4.2 mM MTT, 40 mM EDTA, 1.67 mM PES, and substrate (either 5 M ethanol or 25 mM glucose-6-phosphate). After addition of the appropriate reaction mixture (ethanol for NAD(H), glucose-6-phosphate for NADP(H)), the plate was incubated at 37°C for 5 minutes. To start the reaction, either 10 units/ml alcohol dehydrogenase (from 100 units/ml stock) or 0.27 units/ml glucose-6-phosphate dehydrogenase (from 2.7 units/ml stock) was added. The formation of reduced MTT was monitored using a SpectraMax^384 ^plate reader, taking readings every 15 seconds for 10 minutes using a wavelength of 570 nm while being incubated at 37°C. The cofactor concentration of the samples was interpolated by comparing the rate of reaction to that observed in a concentration curve run on the same plate, and subtracting the rate from the background of the sample (reaction without enzyme).

## Competing interests

The authors declare that they have no competing interests.

## Authors' contributions

RK and JWC performed the experiments, DG assisted in experimental design and data analysis, RK and PCC wrote the manuscript. All authors have read and approved the final manuscript.

## Note

**NOTE: Average signal values start at column Q in Table S1 and at column D in Tables S2-S7. Expression ratios start at column H in Tables S2-S7.

## Supplementary Material

Additional file 1 Table S1. Signal values for the complete probe set data for the *E. coli *K-12 genome. Click here for file

Additional file 2 Table S2. Expression levels of the genes that are differentially expressed between WT G and WT conditions (presented in Figure 1). Genes highlighted in green have a CRP binding site close to their start codon. Click here for file

Additional file 3 Table S3. Expression levels of the genes that are differentially expressed between CRP* G and CRP* conditions (presented in Figure 2). Genes highlighted in green have a CRP binding site close to their start codon. Click here for file

Additional file 4 Table S4. Expression levels of the genes that respond differently to the presence of glucose in W3110 compared to PC05, (CRP* G/CRP*)/(WT G/WT) (presented in Figure 3). Click here for file

Additional file 5 Table S5. Expression levels of the genes that are differentially expressed between CRP* G and WT G conditions (presented in Figure 4). Click here for file

Additional file 6 Table S6. Expression levels of the genes that are differentially expressed between CRP* and WT conditions. Click here for file

Additional file 7 Table S7. Expression levels of the genes that are differentially expressed between CRP* G and WT conditions. Click here for file

Additional file 8 Table S8. a) Primer and probe sequences used for RT-PCR. b) Comparison between microarray and real-time reverse transcription PCR results. Data are presented as fold changes (signal ratio) for all conditions tested. Click here for file

Additional file 9 The file entitled "sup_code.doc" contains the written code in R, used for data analysis. Click here for file
