# Supplementary material for: Transcriptional effects of CRP* expression in Escherichia coli
Source: J Biol Eng. 2009 Aug 24;3:13. doi: 10.1186/1754-1611-3-13 (PMC2743635; doi:10.1186/1754-1611-3-13)
Supplement: Additional file 8 — Table S8. a) Primer and probe sequences used for RT-PCR. b) Comparison between microarray and real-time reverse transcription PCR results. Data are presented as fold changes (signal ratio) for all conditions tested. [file 1754-1611-3-13-S8.doc]

Table S8a. Primers and probes sequences used for RT-PCR

| Primers | Sequence | Probe sequence |
| --- | --- | --- |
| pntARB | TAATTAGCTTCGGCGCTGGTT | CACCACCGCGCTTATTCCAGGC |
| pntAF | GGCAAAAGAGGTCGATATCATTG |  |
| rpsQFB | TGTTGCTATCGAACGTTTTGTGA | CACCCGATCTACGGTAAATTCATCAAGCG |
| rpsQRB | CGTCATGTACGTGCAGTTTGG |  |
| ppsF | TGTTTCCGTTCCGAATGGTT | ACCGCCGACGCGTTTAACCAGTTTC |
| ppsR | TGGTTTACGCCGCTTTGG |  |
| rrsHF | CTACGGGAGGCAGCAGTG | TGSACAATGGGCGMAAGCCTG |
| rrsHR | TGCCAGCAGCCGCGGTAATAC |  |

Table S8b. Comparison between microarray and real-time reverse transcription PCR results. Data are presented as fold changes (signal ratio) for all conditions tested.

|  | *ppsA* | |  | | *pntA* | |  | | *rpsQ* | |
| --- | --- | --- | --- | --- | --- | --- | --- | --- | --- | --- |
| Ratio | Microarray | RT-PCR | | Microarray | | RT-PCR | | Microarray | | RT-PCR |
| WT G / WT | 0.04 | 0.03 | | 0.57 | | 0.41 | | 0.51 | | 0.27 |
| CRP* G / CRP* | 0.38 | 0.26 | | 1.04 | | 1.08 | | 1.22 | | 1.97 |
| CRP* G / WT G | 5.36 | 4.00 | | 1.87 | | 1.51 | | 1.47 | | 1.10 |
