# Supplementary material for: Transcriptional effects of CRP* expression in Escherichia coli
Source: J Biol Eng. 2009 Aug 24;3:13. doi: 10.1186/1754-1611-3-13 (PMC2743635; doi:10.1186/1754-1611-3-13)
Supplement: Additional file 9 — The file entitled "sup_code.doc" contains the written code in R, used for data analysis. [file 1754-1611-3-13-S9.doc]

setwd("C:/Documents and Settings/khankal/My Documents/project 051908/rwork/Ecolik12")

crp <- read.csv("input.csv",header=T)

dim(crp)

crp[1,]

library(limma)

y <- as.matrix(crp[,3:16])

dim(y)

y10<-log(y,base=10)

targets <- c(rep('A',5),rep('B',3),'C','D','D','C','D','C')

f<-factor(targets,levels=c('A','B','C','D'))

design<-model.matrix(~0+f)

fit <- lmFit(y10,design)

contrast.matrix<-makeContrasts((fA-fC),levels=design)

fit3 <- contrasts.fit(fit,contrast.matrix)

fit4 <- eBayes(fit3)

p.values <- fit4$p.value

ids <- as.character(crp[,2])

p.val<-p.adjust(p.values[,1],method="BH")

ord<-order(p.val)

ord.sign<-subset(ord,p.val[ord]<0.05)

ids.sign<-ids[ord.sign]

write.table(ids.sign,file="studyset.txt",sep=" ",col.names=F,row.names=F,quote=F)

y3<-y[ord.sign, ]

aa<-cbind(ids.sign,y3)

write.table(aa,file="aa.txt",sep=" ",col.names=F,row.names=F,quote=F)

results<-decideTests(fit4)

vennDiagram(results)
